# Persistent or permanent atrial fibrillation is associated with severe cardioembolic stroke in patients with non-valvular atrial fibrillation

**DOI:** 10.1186/s12959-021-00276-9

**Published:** 2021-03-31

**Authors:** Joji Hagii, Norifumi Metoki, Shin Saito, Hiroshi Shiroto, Satoko Sasaki, Koki Takahashi, Hiroyasu Hitomi, Yoshiko Baba, Natsumi Yamada, Satoshi Seino, Takaatsu Kamada, Takamitsu Uchizawa, Taigen Nakamura, Minoru Yasujima, Hirofumi Tomita

**Affiliations:** 1 Hirosaki Stroke and Rehabilitation Center, Hirosaki, 036-8104 Japan; 2 Department of Cardiology, Hirosaki University Graduate School of Medicine, 5 Zaifu-cho, Hirosaki, 036-8562 Japan; 3 Department of Stroke and Cerebrovascular Medicine, Hirosaki University Graduate School of Medicine, Hirosaki, 036-8562 Japan

**Keywords:** Paroxysmal atrial fibrillation, Persistent/permanent atrial fibrillation, Cardioembolic stroke, Stroke severity, Underuse

## Abstract

**Background:**

Little is known about the difference in the severity of cardioembolic (CE) stroke between patients with paroxysmal atrial fibrillation (PAF) and persistent/permanent AF (PerAF). We assessed stroke severity in patients with CE stroke divided by the type of AF.

**Methods:**

Three hundred and fifty-eight consecutive patients with CE stroke within 48 h of onset and with a modified Rankin Scale (mRS) score ≤ 1 before onset were studied. We compared basic characteristics, stroke severity, and functional outcome between patients with PAF (*n* = 127) and PerAF (*n* = 231).

**Results:**

Patients with PerAF were more likely to take oral anticoagulants (OACs) than those with PAF (37% vs. 13%, *P* <  0.0001), even though still underuse of OAC in both patients. Regarding stroke severity on admission, patients with PerAF exhibited a tendency toward a higher score on the National Institutes of Health Stroke Scale (NIHSS) compared with patients with PAF (12 [5–20] vs. 9 [4–18]; *P* = 0.12). Mortality and mRS score at discharge were higher in the PerAF than in the PAF group (13% vs. 4%; *P* = 0.005, and 3 [1–5] vs. 2 [1–4]; *P* = 0.01, respectively). Multivariate analyses confirmed that PerAF was a significant determinant of severe stroke (NIHSS score > 8) on admission (odds ratio [OR] to PAF = 1.80; 95% confidence interval [CI] 1.08–2.98; *P* = 0.02) and of an mRS score ≥ 3 at discharge (OR = 2.07; 95% CI 1.24–3.46; *P* = 0.006). Patients with PerAF had three times more internal carotid artery occlusion evaluated by magnetic resonance angiography, which indicated a more severe cerebral embolism compared with patients with PAF.

**Conclusions:**

We found underuse of OAC in high risk AF patients with CE stroke. PerAF is significantly associated with severe stroke on admission and an unfavorable functional outcome at discharge in Japanese patients with CE stroke.

## Background

Cardioembolic (CE) stroke has a poor functional prognosis compared with other types of cerebral infarction [1, 2]. The aging of the Japanese population is accompanied by increases in the rate of atrial fibrillation (AF) and CE stroke. Regardless of the type of AF, the current guidelines suggest that risk assessment should be performed for paroxysmal AF (PAF) and persistent or permanent AF (PerAF) equally and that anticoagulation therapy should be performed accordingly [3]. Direct oral anticoagulants (DOACs), including dabigatran, rivaroxaban, apixaban, and edoxaban, are used widely as first-line drugs for the prevention of stroke and systemic thromboembolism in patients with non-valvular AF (NVAF).

To date, the type of AF has not been taken into consideration in this setting. Although a previous study showed that PAF and PerAF have no effect on the risk of stroke [3], recent studies have shown that PAF is linked to a lower incidence of stroke compared with PerAF [4–7]. Moreover, it has been reported that Japanese patients with PAF have a more favorable clinical outcome than those with PerAF after CE stroke [8].

In the present study, we compared CE stroke severity on admission and functional outcome at discharge between patients with PAF and PerAF.

## Methods

### Study patients

The Hirosaki Stroke and Rehabilitation Center (HSRC) has both a stroke care unit for acute therapy and a stroke rehabilitation unit for further rehabilitation therapy. Therefore, all patients with acute ischemic stroke admitted to HSRC receive consistent therapies in the acute phase, and subsequently in the chronic phase during hospitalization.

**Fig. 1:**
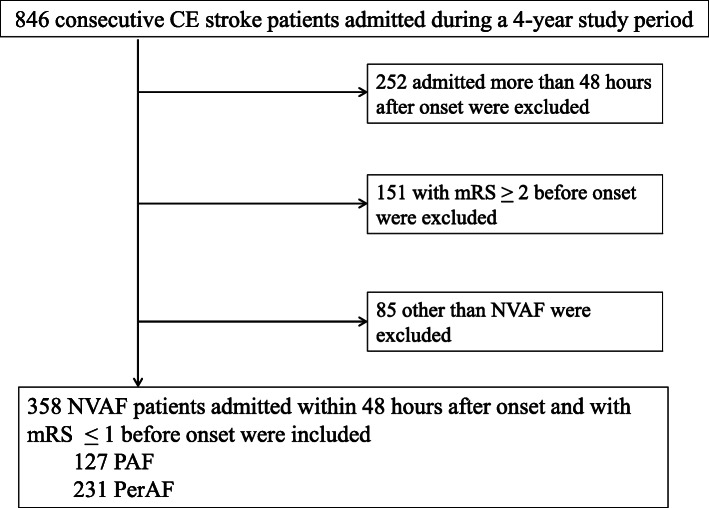
Flowchart of the patient selection procedure. CE stroke indicates cardioembolic stroke, mRS; modified Rankin Scale, NVAF; non-valvular atrial fibrillation, PAF; paroxysmal atrial fibrillation, PerAF; persistent or permanent atrial fibrillation

Over the 4-year period from April 2011 to March 2015, a total of 846 consecutive patients with CE stroke were admitted to the HSRC for acute therapy and further rehabilitation within 60 days after the onset of CE stroke. Among them, 358 patients with NVAF who were admitted to the HSRC within 48 h of the onset of stroke and had a modified Rankin Scale (mRS score) of 0 or 1 (i.e., without any limitation in physical activities) before the onset of stroke were included in the present study (Fig. 1). The clinical characteristics, stroke severity on admission, and functional outcome at discharge were compared between patients with PAF (*n* = 127) and PerAF (*n* = 231).

According to the guidelines of the European Society of Cardiology [9], AF is classified into PAF that returns to sinus rhythm spontaneously within 7 days (usually within 48 h), persistent AF that lasts longer than 7 days but can be returned to sinus rhythm by treatment, long-standing persistent AF that lasts longer than 1 year but can be considered to return to sinus rhythm, and permanent AF that is accepted by the patient and physician, and no further attempts to restore/maintain sinus rhythm. In the present study, the type of AF was determined either as a PAF or as a PerAF that included persistent AF, long-standing persistent AF, and permanent AF. Patients with AF that returned to sinus rhythm spontaneously within 2 weeks in the acute phase of stroke treatment were also classified as a PAF.

The subjects were registered in the Hirosaki Stroke Registry (UMIN Clinical Trials Registry: UMIN000016880).

### Diagnosis, stroke severity, and outcome

All patients underwent brain computed tomography on admission. When intracerebral hemorrhage was not detected, we further performed brain magnetic resonance imaging including transversal diffusion-weighted imaging, T2-weighted imaging, fluid-attenuated inversion recovery, and magnetic resonance angiography (Signa Excite HD 1.5 T; GE Medical System, Waukesha, WI). Carotid ultrasonography, chest X-ray, 12-lead and 24 h Holter electrocardiogram (ECG), and standard blood tests were performed on all patients. If necessary, an ECG monitor was installed for 2 weeks during the acute treatment phase of stroke and transesophageal echocardiography was performed. CE stroke was diagnosed according to the Trial of Org10172 in Acute Stroke Treatment Classification [10].

Thrombolysis therapy with intravenous recombinant tissue plasminogen activator (rt-PA) was performed according to the Japanese Guideline [11]. Treatment with OACs before onset was also assessed. Stroke severity was assessed based on National Institutes of Health Stroke Scale (NIHSS) score on admission. Severe stroke was defined as an NIHSS score ≥ 8 [12, 13]. The mRS score at discharge was evaluated as a measure of functional outcome. The CHADS_2_ and CHA_2_DS_2_-VASc scores, for risk stratification of thromboembolism before stroke onset, and the HAS-BLED score, for bleeding risk stratification before stroke onset, were also determined in each patient, as described previously [9, 13–15]. The risk factors were as follows: congestive heart failure (left ventricular ejection fraction < 40%, New York Heart Association class II or higher heart failure symptoms within 6 months before stroke onset), hypertension (treatment with antihypertensive medication or documented systolic blood pressure ≥ 140 mmHg or diastolic blood pressure ≥ 90 mmHg), diabetes mellitus (treatment with insulin or antidiabetic medication, or at least two determinations of diabetic type on separate days as evaluated by an oral glucose tolerance test, fasting blood glucose ≥126 mg/dL, casual blood glucose ≥200 mg/dL, or HbA1c ≥6.5%), vascular disease (coronary artery disease, ankle brachial index ≤0.9, or aortic plaque), and dyslipidemia (treatment with lipid-lowering medication, low-density lipoprotein cholesterol ≥140 mg/dL, high-density lipoprotein cholesterol < 40 mg/dL, or triglycerides ≥150 mg/dL).

### Statistical analysis

Data were expressed as the median (25th–75th percentiles) or n (%). A Mann–Whitney *U* test, chi-squared test, or Fisher’s exact test was used to compare differences between two groups, as appropriate. The effects of AF type on the NIHSS score on admission and the mRS score at discharge were assessed by multivariate logistic regression analysis as an odds ratio (OR) adjusted for age, sex, body mass index (BMI), congestive heart failure, hypertension, diabetes mellitus, prior cerebral infarction or transient ischemic attack, vascular disease, serum creatinine (Cre) level, and OAC treatment. All statistical analyses were performed using the JMP Pro 11 software (SAS, Cary, NC). Significance was set at *P* <  0.05.

## Results

### Patient profiles

A comparison of the clinical characteristics of the patients with PAF and PerAF in this study is shown in Table 1. The median age, BMI, and proportion of males were similar between the two groups. The CHADS_2_ score, CHA_2_DS_2_-VASc score, and HAS-BLED score were significantly higher in the PerAF than in the PAF group. Congestive heart failure, diabetes mellitus, and prior cerebral infarction or transient ischemic attack were more frequent in the PerAF than in the PAF group. The rates of hypertension, vascular disease, antiplatelet use, smoking, and dyslipidemia did not differ between the two groups. Cre and Cre clearance (CCr) were similar between the two groups. The proportion of patients undergoing OAC therapy before stroke onset was higher in the PerAF group than in the PAF group, even though still underuse of OAC in both groups. There were no differences between the two groups regarding the proportion of patients treated with rt-PA thrombolysis.

**Table 1:** Clinical characteristics of the study patients

	PAF (*n* = 127)	PerAF (*n* = 231)	*p*-value
Basic characteristics			
Age (years)	80 (73–84)	79 (73–84)	0.94
Male gender	66 (52%)	128 (55%)	0.53
BMI (kg/m^2^)	22.6 (20.6–24.9)	22.5 (20.5–25.2)	0.73
Risk stratification			
CHADS_2_ score	3 (2–4)	3 (2–4)	< 0.0001
CHA_2_DS_2_-VASc score	4 (3–5)	5 (4–6)	< 0.0001
HAS-BLED score	3 (2–3)	3 (2–4)	0.0004
Congestive heart failure	32 (25%)	92 (40%)	0.005
Hypertension	95 (75%)	180 (78%)	0.52
Diabetes mellitus	27 (21%)	80 (35%)	0.008
Prior cerebral infarction or TIA	45 (35%)	121 (52%)	0.003
Vascular disease	23 (18%)	63 (27%)	0.05
Antiplatelet use	26 (20%)	54 (23%)	0.60
Smoking	14 (11%)	32 (14%)	0.51
Dyslipidemia	78 (61%)	140 (61%)	0.91
Blood chemistry			
Cre (mg/dL)	0.83 (0.72–0.99)	0.85 (0.71–1.11)	0.32
CCr (mL/min)	51.6 (38.8–65.2)	50.3 (37.3–66.7)	0.83
PT-INR	0.99 (0.92–1.05)	1.04 (0.96–1.15)	< 0.0001
Treatment			
rt-PA thrombolysis	22 (17%)	33 (14%)	0.45
OAC before onset			< 0.0001
None	110 (87%)	145 (63%)	
WF	11 (9%)	72 (31%)	
DOAC	6 (5%)	14 (6%)	

Data are shown as median (25th–75th percentiles) or n (%). Creatinine clearance was estimated by the Cockcroft-Gault equation. PAF indicates paroxysmal atrial fibrillation, *PerAF* persistent or permanent atrial fibrillation, BMI; body mass index, TIA; transient ischemic attack, *Cre* serum creatinine, *CCr* creatinine clearance, *PT-INR* prothrombin time-international normalized ratio, *rt-PA* recombinant tissue plasminogen activator, *OAC* oral anticoagulant, *WF* warfarin, *DOAC* direct oral anticoagulant

### Comparisons of stroke severity on admission and functional outcome at discharge

**Fig. 2:**
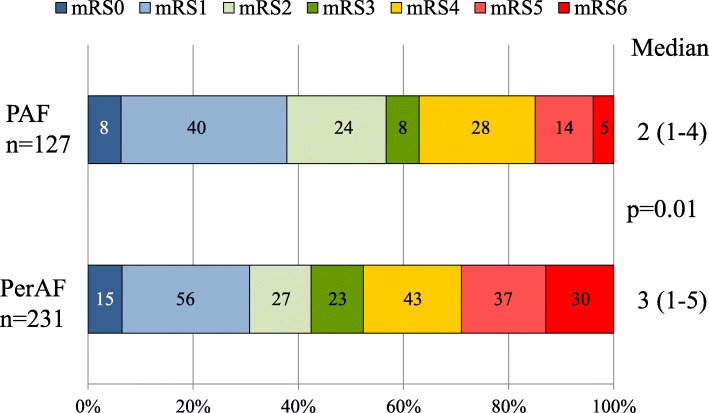
Comparison of the modified Rankin Scale (mRS) score at discharge between patients with PAF and PerAF. The median value and 25th–75th percentiles are shown in each group. The numbers in the graphs indicate the number of patients. PAF indicates paroxysmal atrial fibrillation, PerAF; persistent or permanent atrial fibrillation

Stroke severity on admission was assessed using the NIHSS. The NIHSS score tended to be higher in the PerAF group than in the PAF group, although not statistically significant (12 [5–20] vs. 9 [4–18]; *P* = 0.12) (Table 2). Functional outcome at discharge, as assessed by mRS score, was unfavorable in the PerAF group compared with the PAF group (3 [1–5] vs. 2 [1–4]; *P* = 0.01 (Fig. 2). Consistently, mortality was significantly higher in the PerAF group than in the PAF group (13% vs. 4%; *P* = 0.005) (Fig. 2 and Table 2).

**Table 2:** Comparisons of stroke severity on admission and outcomes at discharge between PAF and PerAF patients

	PAF (n = 127)	PerAF (n = 231)	p-value
Stroke severity			
NIHSS on admission	9 (4–18)	12 (5–20)	0.12
Mortality	5 (4%)	30 (13%)	0.005

Data are shown as median (25th–75th percentiles) or n (%). PAF indicates paroxysmal atrial fibrillation, *PerAF* persistent or permanent atrial fibrillation, *NIHSS* the National Institutes of Health Stroke Scale

A multivariate logistic regression analysis showed that PerAF was a significant determinant of severe stroke (NIHSS score ≥ 8) on admission (OR to PAF = 1.80; 95% confidence interval [CI], 1.08–2.98; *P* = 0.02) (Table 3). PerAF was also a significant determinant of having an mRS score ≥ 3 at discharge (OR to PAF = 2.07; 95% CI, 1.24–3.46; *P* = 0.006) (Table 3).

**Table 3:** Multivariate logistic regression analysis for stroke severity on admission and outcomes at discharge

	Odds ratios	95% CI	*p*-value
NIHSS score on admission (> 8)			
PAF	Reference		
PerAF	1.80	1.08–2.98	0.02
mRS score at discharge (> 3)			
PAF	Reference		
PerAF	2.07	1.24–3.46	0.006

Odds ratios were calculated using PAF patients as a reference after adjustment for age, sex, body mass index, congestive heart failure, hypertension, diabetes mellitus, prior cerebral infarction or transient ischemic attack, vascular disease, serum creatinine, and oral anticoagulant treatment. CI indicates confidence interval, *PAF* paroxysmal atrial fibrillation, *PerAF* persistent or permanent atrial fibrillation, *NIHSS* the National Institutes of Health Stroke Scale, *mRS* modified Rankin Scale

### Evaluation of internal carotid artery occlusion on admission

**Fig. 3:**
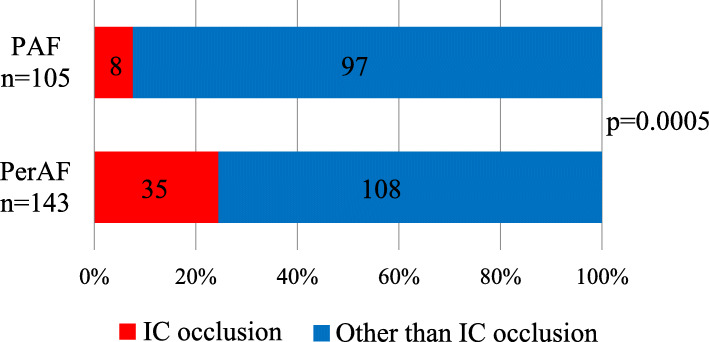
Percentage of internal carotid artery occlusion in cardioembolic stroke. Only cases in which MRI could be performed and those without anticoagulation therapy before stroke onset were included. The numbers in the graphs indicate the number of the patients. PAF indicates paroxysmal atrial fibrillation, PerAF; persistent or permanent atrial fibrillation, ICA; internal carotid artery

Internal carotid artery occlusion, which is indicative of severe cerebral embolism, was examined by MR angiography on admission in patients without OAC therapy before stroke onset (PAF, *n* = 105 and PerAF, *n* = 143). The proportion of internal carotid artery occlusion was higher in the PerAF group than in the PAF group (35/143 (24%) vs. 8/105 (8%); *P* = 0.0005) (Fig. 3).

## Discussion

In the present study, we found that the type of AF was related to the severity of CE stroke, as PerAF yielded a more severe outcome than PAF. PerAF was significantly associated with severe stroke on admission and an unfavorable function outcome at discharge. The increased rate of internal carotid artery occlusion found in patients with PerAF may be responsible for the more severe stroke observed in these patients.

Although the specific mechanism by which the severity of stroke differed depending on the type of AF is unknown, it may be explained by differences in the mechanism of thrombus formation. In studies using echocardiography, left atrial appendage (LAA) blood flow and left atrial diameter differ between patients with PAF and PerAF; patients with PerAF exhibits a lower LAA flow velocity, larger left atrial diameter, and even more frequent spontaneous echo contrast [16–18]. CE stroke is likely to occur in the presence of a low flow velocity or spontaneous echo contrast in the LAA [19, 20]. Therefore, it is expected that PerAF is more likely to cause thrombosis than PAF. This seems to explain the observation that patients with PAF exhibit a lower incidence rate of stroke compared with patients with PerAF.

In addition, it is expected that the size and hardness of the thrombus are different between the two groups and are related to the severity of CE stroke. It can be imagined that a soft little thrombus is easy to break apart, even if the vessel is occluded, and that it is easy to reopen the blood vessel. Conversely, the blockage of the relatively thick artery of the neck or the brain by a large hard thrombus leads to serious cerebral infarction. The thickest blood vessel that can be obstructed in cardiogenic cerebral embolism is the internal carotid artery. In cases in which anticoagulant therapy was not received before the onset of stroke and MRI was used to image the lesion, we compared cases of internal carotid artery occlusion in the two groups (PerAF and PAF) and found that patients with PerAF had three times more internal carotid artery occlusion than those with PAF. This suggests that PerAF exhibits thrombi that are bigger and harder than PAF. Koga et al. reported that stroke severity and short-time functional outcomes could also be attributable to the thrombus-formation tendency of the LAA [6]. Because morphological and functional differences in the LAA between the two groups may affect cardiac embolus size and the hardness or resistance to thrombolysis, they may affect the incidence of internal carotid artery occlusion, and consequently influence on stroke severity and functional outcome. More evidence regarding this issue is required.

Several recent studies reported that patients with PerAF have a higher incidence of stroke than those with PAF [4–7], and our study further showed that patients with PerAF have a worse prognosis than those with PAF. Therefore, it is of significant importance to avoid the progression of PAF to PerAF. Age, hypertension, heart failure, chronic kidney disease, diabetes mellitus, physical inactivity, obesity, and enlarged left atrial size are known to be risk factors for AF progression [21, 22]. Accordingly, a structured and comprehensive risk factor management program focusing on modifiable factors such as hypertension, physical inactivity, and obesity may be effective in preventing AF progression. Furthermore, it is very meaningful to identify AF at an early stage (i.e., PAF) before progression to PerAF. Opportunistic screening for AF by pulse taking or ECG rhythm strip is also useful and recommended in individuals ≥65 years of age [22].

Underuse of OAC in elderly NVAF patients is a major issue in the aging countries such as Japan. The Fushimi AF Registry, one of the Japanese AF registries, reported that almost 60% of extreme elderly patients (≥ 85 years old) did not take OACs. The present study also showed that 87% of PAF and 63% of PerAF patients with a median age of 79–80 years old did not have OAC therapy, even though relatively high median CHADS_2_ score of 3 (Table 1). As extreme elderly NVAF patients are at high risk for the incidence of ischemic stroke [7], the guideline-based adequate OAC therapy is considered and recommended after careful assessment of bleeding risk, cognitive state, mobility, frailty, and socioeconomic factors. Recent phase 3 randomized double-blind study to compare a once-daily edoxaban 15 mg with placebo in Japanese patients 80 years of age or older demonstrated that edoxaban 15 mg was superior to placebo in preventing stroke or systemic embolism and did not have a significantly higher incidence of major bleeding than placebo [23]. Furthermore, multicenter prospective observational studies of Japanese NVAF patients aged ≥75 years have been conducted [24, 25]. The results of these studies will provide an important clinical implication for clinical practice in elderly NVAF patients.

The analyses performed in this study have several limitations. First, our study was a single-center, observational one; therefore, generalization of our results may be limited. However, we studied consecutive patients who were admitted during the study period and analyzed the severity and functional outcome of all patients, thereby likely minimizing the biases caused by an observational study. Second, we studied only the internal carotid artery as an occlusion site in patients with CE stroke by MR angiography; thus, other sites and sizes of cerebral infarction were not examined. However, the severity of cerebral infarction cannot be determined based only the size of the infarct. Furthermore, severity will change if recanalization of the obstructed blood vessel accompanies the hemorrhagic infarction. It is quite difficult to evaluate these situations in detail. Third, we classified the type of AF into only PAF and PerAF, and did not evaluate AF burden in detail. Furthermore, as we included patients as a PAF when AF returned to sinus rhythm spontaneously within 2 weeks in the acute phase of stroke treatment, some patients with persistent AF may have been included in the PAF group. Finally, the precise mechanism by which the CE stroke caused by PAF is favorable compared with that caused by PerAF remains largely unknown.

## Conclusions

Underuse of OAC in high risk patients with CE stroke is still a major concern in clinical setting. The type of AF is related to the severity of CE stroke, with PerAF being more severe than PAF. PerAF in patients with CE stroke is a significant predictor of severe stroke on admission and unfavorable function outcome at discharge, partly through internal carotid artery occlusion.

## Data Availability

The datasets used and/or analyzed during the current study are available from the corresponding author on reasonable request.

## Abbreviations

AF: Atrial fibrillation
BMI: Body mass index
CCr: Creatinine clearance
CE: Cardioembolic
CI: Confidence interval
Cre: Creatinine
DOAC: Direct oral anticoagulant
ECG: Electrocardiogram
HSRC: The Hirosaki Stroke and Rehabilitation Center
LAA: Left atrial appendage
mRS: Modified Rankin Scale
NIHSS: The National Institutes of Health Stroke Scale
NVAF: Non-valvular atrial fibrillation
OAC: Oral anticoagulant
OR: Odds ratio
PAF: Paroxysmal atrial fibrillation
PerAF: Persistent/permanent atrial fibrillation
rt-PA: Recombinant tissue plasminogen activator
